# Transanal intersphincteric under direct view in the jackknife position combined with laparoscopic total mesorectal excision for treating ultra-low rectal cancer

**DOI:** 10.3389/fsurg.2024.1419675

**Published:** 2024-09-16

**Authors:** Guobin Zhong, Zhiyu Chen, Zhenfeng Li, Bin Zhao, Junhui Deng

**Affiliations:** ^1^ Department of Radiotherapy, Huizhou Municipal People’s Hospital, Huizhou, China; ^2^ Department of Colorectal and Anal Surgery, Huizhou Municipal People's Hospital, Huizhou, China

**Keywords:** jackknife position, intersphincteric, the level of the levator anal hiatus, ultra-low rectal cancer, total mesorectal excision

## Abstract

**Aim:**

To investigate the effect and clinical advantage of transanal intersphincteric (ISR) under direct view in the jackknife position combined with laparoscopic total mesorectal excision (TME) for treating ultra-low rectal cancer. Additionally, the feasibility of this surgical technique was evaluated.

**Method:**

This was a retrospective, single-center, single-arm pilot study. Ten patients with ultra-low rectal cancer underwent treatment by the same surgical team for direct view transanal ISR combined with laparoscopic TME in the Department of Anorectal Surgery, Huizhou Central People's Hospital between January 2021 and June 2021. The relevant clinical data were collected and analyzed.

**Results:**

All the patients underwent complete mesenteric resection without conversion to laparotomy. The circumferential and distal resection margins (CRM and DRM) were negative. The mean distance between the lower margin of the tumor and the anal margin was 2.8 ± 0.8 cm, and the mean margin of distal resection was 1.2 ± 0.2 cm. TNM pathological stages I, II, III, and IV were observed in 6, 2, 2, and 0 cases, respectively. The median follow-up period was 15 months (interquartile range, 8 months). The mean Wexner and Low Anterior Resection Syndrome scores at 12 months after ileostomy were 8.1 ± 2.1 and 22.4 ± 5.7, respectively.

**Conclusion:**

Transanal ISR under direct view in the jackknife position combined with laparoscopic TME is safe and feasible for the treatment of ultralow rectal cancer.

## Introduction

Rectal cancer is one of the most common malignant tumors worldwide and is associated with high morbidity and mortality (1). According to the distance between the rectal tumor and the dentate line, rectal cancers can be classified as high, middle, low, and even ultralow. In China, the proportion of patients with low- and ultra-low rectal cancer is as high as 60%–70% (2). Although new advances have been made in rectal cancer treatment in recent years, surgery remains the key treatment, especially for early-stage patients. Surgeons consistently face the challenge of not only achieving complete tumor removal, but also preserving the anal function of the patient as much as possible.

## Methods

### Patient enrollment

We retrospectively analyzed the clinical cases of 10 patients with ultralow rectal cancer who underwent transanal intersphincteric (ISR) combined with laparoscopic total mesorectal excision (TME) at the Department of Anorectal Surgery of Huizhou Municipal People's Hospital between January 2021 and June 2021. The same surgical team treated all patients.

Patients were included according to the following criteria: (1) Aged 18–80 years; (2) Colonoscopy, abdominal magnetic resonance imaging and digital rectal examination were performed before surgery, and it was confirmed that the lowest edge of the tumor was less than 3 cm above anal verge; (3) Pathological biopsy was used to confirm the presence of highly or moderately differentiated rectal cancer; (4) Preoperative computed tomography excluded distant metastasis, and the clinical stage was T1–3NxM0; (5) Good preoperative anal control function was recorded; and the patient had a strong desire to preserve the anus. (6) All patients and their families provided written informed consent and completed the postoperative follow-up. Patients were excluded based on the following criteria: (1) Presence of delirium, inability to communicate effectively, or mental impairment; (2) Preoperative evaluation revealed coagulation dysfunction, pulmonary infection, severe pulmonary insufficiency, and severe cardiovascular and cerebrovascular diseases; (3) Administration of neoadjuvant radiotherapy; (4) Presence of combined intestinal obstruction, intestinal perforation, or colorectal multisource cancer; and (5) History of abdominal, pelvic, or anal surgery and other tumors.

### Surgical procedure

**Figure 1:**
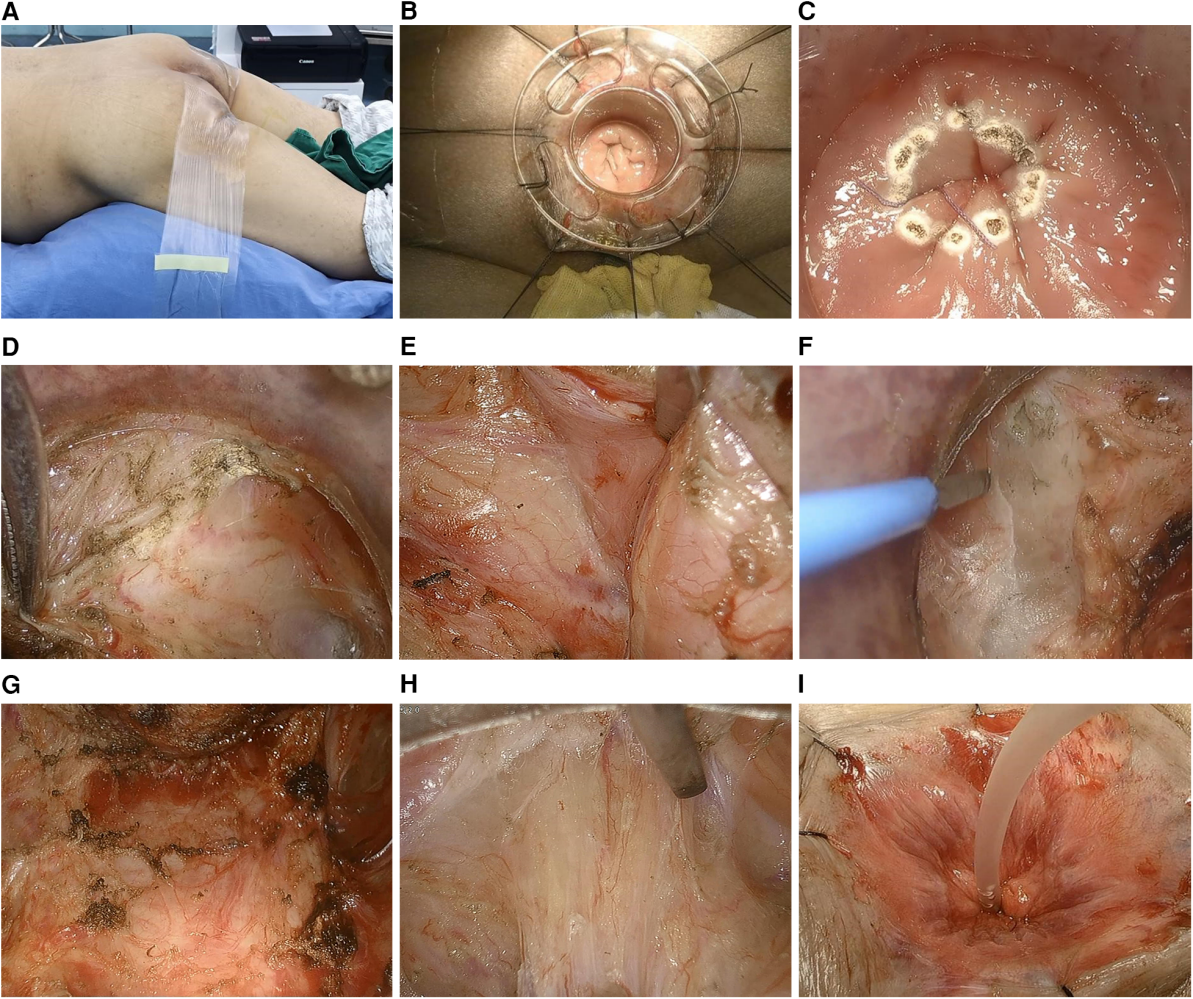
**(A)** Jackknife position. **(B)** Implantation of anal reaming device. **(C)** Purse-string suture isolated the tumor and marked the incision margin. **(D)** Incision of the longitudinal rectal muscle. **(E)** Separation of the right lateral wall of the internal and external anal sphincter space. **(F)** Separation of the left lateral wall of the internal and external anal sphincter space. **(G)** Separation of the anterior rectal wall (rectourethral muscle). **(H)** Separation of the posterior rectal wall. **(I)** The broken end of the anal tube was sewed with a purse-string and the anastomotic guide tube was inserted.

The specific surgical procedures involved in transanal ISR resection under direct view in the prone jackknife position and laparoscopic TME were as follows. First, transanal ISR resection was performed under direct view in the jackknife position. After endotracheal intubation under general anesthesia, the patient was first placed in the jackknife position. The anal retractor was then placed to completely expand the anus. The tumor's lower margin was measured and located at least 0.5 cm below the purse-string. A purse-string suture was performed to close the intestinal cavity, and the incision margin, cut with an electroknife, was located approximately 0.5–1 cm below the purse-string. After the annular incision of the submucosa, the procedure advanced into the plane of the muscularis propria of the rectum, with continued lateral incision of the annular muscle. An incision in the medial longitudinal muscle penetrated the internal and external sphincter spaces. If the incision is excessively deep and the external sphincter is injured, muscle contraction occurs, which should be promptly corrected to ensure free plane between the internal and external sphincters. The free sphincter space was elevated 2–3 cm to the level of the hiatus of the anal extensor muscle. The anterior and posterior rectal walls were successively separated. A small gauze was inserted to mark the subsequent abdominal operation. The broken end of the anal tube was sewed with a purse-string, and the anastomotic guide tube was inserted (Figure 1).

**Figure 2:**
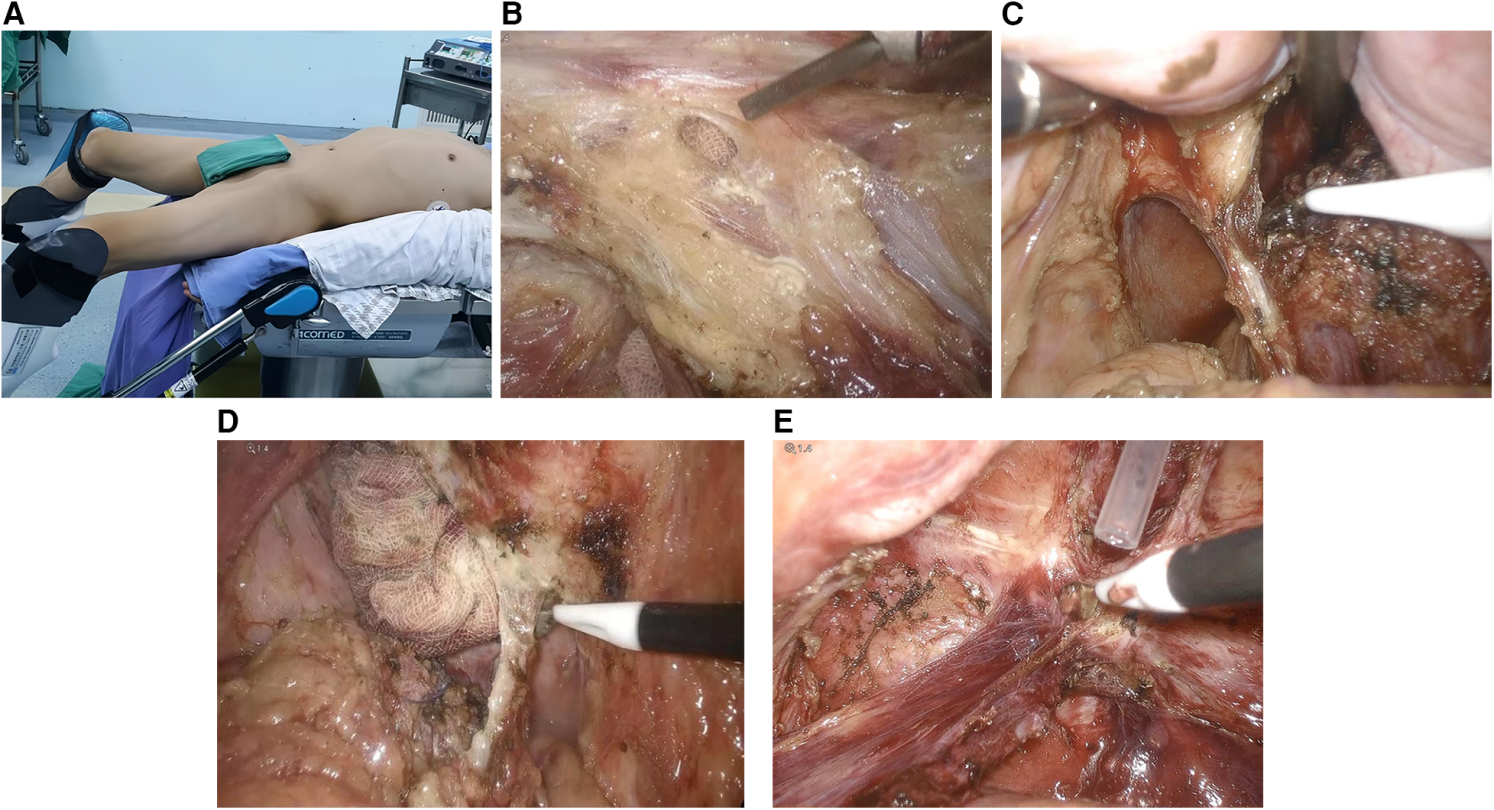
**(A)** Improved lithotomy position. **(B)** Incision of the anterior rectal wall to reveal the transanal insertion of the gauze. **(C)** Cut the left hiatus ligament. **(D)** Cut the right hiatus ligament. **(E)** Cut the anococcygeal ligament.

Second, modified lithotomy position transabdominal laparoscopic TME was performed. The modified lithotomy position was changed, and abdominal surgery was performed using the five-hole method. Following the principles of tumor surgical exploration and TME, the left half mesocolon and splenic flexure were freed laparoscopically, the arteriovenous of the lower mesocolon was ligated and cut off at a high level, the anterior or lateral rectal wall was exposed to find the sphincter space, and the rectum was pulled during the transanal operation. The lateral hiatus ligament was dissociated, and the anococcygeal ligament was finally severed in the posterior wall of the rectum (Figure 2).

Finally, transanal anastomosis was performed. The specimen was removed through the anus, the mesentery was cut, and the specimen was excised. The distal resection margin was determined to be negative via rapid freezing pathology. The pneumoperitoneum was reconstructed, and transanal implantation using a 28-mm stapler was performed to complete transanal anastomosis with the proximal colon. Lastly, the anastomosis was strengthened by transanal suturing, if necessary. All patients underwent prophylactic ileostomy.

### Statistical analysis

IBM SPSS Statistics (version 25.0) software (IBM Corp, Armonk, NY, USA) was used for statistical analysis. Data normality was assessed using the Shapiro-Wilk test. Normally distributed data were expressed as the mean ± standard deviation, whereas non-normally distributed data were expressed as the median [interquartile distance (IQR)]. The *t*-test was used to compare two independent samples, and *P* < 0.05 was considered statistically significant.

## Result

The clinical characteristics of the patients are summarized in Table 1. The patients (6 males and 4 females), had a mean age of 67.2 ± 7.8 years and a mean body mass index of 22.8 ± 2.6 kg/m^2^. According to the American Society of Anesthesiologists Physical Condition (ASA) classification, 5 patients were classified as ASA Class I and the remaining 5 as ASA Class II. The mean distance between the lower margin of the tumor and the anus was 2.8 ± 0.8 cm. All patients underwent R0 resection, and the surgical resection and anatomical degree met the criteria for radical resection of rectal cancer. No conversion to laparotomy was performed. The mean operation time was 240 ± 28 min, and the mean blood loss was 30 ml (IQR, 20 ml).

**Table 1:** Patients’ demographic and clinical characteristics.

Gender (male/female)	6/4
Mean age, years	67.2 ± 7.8
Mean BMI, kg/m^2^	22.8 ± 2.6
ASA physical status classification	
ASAⅠ	5
ASAⅡ	5
Mean distance of tumour's lower edge from anal verge, cm	2.8 ± 0.8
Mean operation time, min	240 ± 28
Median blood loss, ml(IQR)	30 (20)

The pathological features of the tumors are presented in Table 2. Of the 10 patients, 4 exhibited highly differentiated adenocarcinomas and 6 had moderately differentiated adenocarcinomas. TNM pathological stages I, II, III, and IV were observed in 6, 2, 2, and 0 cases, respectively. The median maximum tumor diameter was 3.0 cm (IQR, 1.2 cm), and the number of lymph nodes was 17.8 ± 4.8. Distal resection margin (DRM) and circumferential resection margin results were negative in all patients, with a mean DRM length of 1.2 ± 0.2 cm.

**Table 2:** Postoperative tumour pathology.

Histological differentiation	Number
Adenocarcinoma	10
high	4
Moderate	6
pT category	
Tis	0
1	5
2	3
3	2
4	0
pN category	
0	8
1	1
2	1
M category	
0	10
1	0
pTNM stage	
Ⅰ	6
Ⅱ	2
Ⅲ	2
Ⅳ	0
Median maximum diameter of tumor, cm(IQR)	3.0（IQR 1.2）
Mean number of nodes	17.8 ± 4.8
Mean distal resection margin, cm	1.2 ± 0.2
Positive circumferential margin	0
Positive distal margin	0
Vascular invasion	1/10
Nerve invasion	2/10

The postoperative recovery of the patients are summarized in Table 3. The duration of initiation of a semi-liquid diet was 4 days (IQR 2 days), the mean length of hospital stay was 9 days (IQR 5 days), and there were no perioperative complications. All patients underwent ileostomy reduction within 3–6 months after surgery. Median follow-up was 15 months (IQR 8 months), and no patient showed signs of local recurrence or distant metastasis. One patient developed grade A anastomotic fistula 30 days after surgery, and two patients developed membranous anastomotic stenosis. Defecation function was evaluated using Wexner's fecal incontinence score and Low anterior resection (LARS) score. Wexner scores were 11.2 ± 3.0 and 8.1 ± 2.1 at 6 and 12 months after surgery, and LARS scores were 28.3 ± 4.6 and 22.4 ± 5.7, respectively. Wexner and LARS scores at 12 months after surgery were significantly lower than those at 6 months after surgery (*P* < 0.05).

**Table 3:** Postoperative recovery.

Median time to liquid food, days(IQR)	4 (2)
Median time to discharge surgery, days(IQR)	9 (5)
Median time to stoma closure, days(IQR)	128 (32)
Median follow-up time, months(IQR)	15 (8)
Wexner score	*P *< 0.05
6 months	11.2 ± 3.0
12 months	8.1 ± 2.1
LARS score	*P* < 0.05
6 months	28.3 ± 4.6
12 months	22.4 ± 5.7

## Discussion

The lesion of ultralow rectal cancer is located deep in the pelvis, which is difficult to reveal due to the limitation of the pelvis and adjacent organs. Wiliams believed that the farthest end of the rectum wrapped area of the levator ANI muscle was difficult for surgeons to reach under direct vision, and was a “no man's land” for rectal surgery (3). At present, the mainstream operations for ultra-low rectal cancer include: transabdominal laparoscopic TME + transanal ISR, laparoscopic transabdominal approach ISR, TaTME and laparoscopically assisted TaTME. All of the above operations are completed at the lithotomy position. In this study, we report the technique and results of direct viewing transanal ISR in the jackknife position combined with laparoscopic TME in treating ultra-low rectal cancer. Due to the deep location of ultralow rectal cancer within the pelvic cavity, a classical method for its treatment involves combined abdominal and perineal resection. However, this surgery cannot preserve the anus and requires permanent colostomy, which seriously reduces the quality of life of patients and is difficult to promote. In 2000, Watanabe reported for the first time that laparoscopic TME combined with transanal ISR could be used for the treatment of ultra-low rectal cancer. With the advantage of laparoscopic field amplification, the operator can achieve a more accurate free space in the narrow pelvic floor space, and the plane of downwards separation is often lower than that of traditional open surgery, reducing the degree of complete mesocentric resection (4). However, transanal ISR during lithotomy and accurate internal and external sphincter dissociation and disarticulation to achieve high-quality terminal rectal resection are difficult to perform.

In 2010, poolside laparoscopic ISR was proposed for the first time (5). During the operation, guided by distinct anatomical marks around the mesorectum, the entire mesorectum was sequentially dissected from top to bottom. This involved following the established planes and spaces from the abdominal operation, reducing damage to the surrounding trachea and achieving the dissociation between sphincter muscles. However, accurately locating and detaching the distal margin of the rectum transabdominally is impossible. Moreover, when the abdomen passes over the pelvis, the visual field gradually narrows, the operation path becomes longer owing to the smaller and limited anatomical space, and the difficulty of the surgery increases significantly, especially in male patients with a narrow pelvis, large tumors, or obesity. Below the level of the levator anal hiatus, it is difficult to distort the sphincter muscles through the abdomen because of the absence of the mesorectal mesentery and lack of antagonizing traction. During surgery, deviations from the correct level pose a risk of rectal perforation or external sphincter injury, potentially affecting the quality of terminal rectum resection. Therefore, this surgery is suitable for patients with early rectal cancer located within 2–5 cm of the dentate line.

In recent years, transanal total mesorectal excision (TaTME) has been used to achieve radical resection of lower rectal cancer while preserving the anus. In contrast to the traditional path, the operation is performed from the bottom up. It has unique advantages in ensuring sufficient distal and circumannular margins of tumors and in reducing the difficulty of distal rectal resection. This aligns with the concept of natural orifice transluminal endoscopic surgery (NOTES), an innovative surgical procedure. Therefore, this has become a research hotspot in the surgical treatment of low rectal cancer. Current studies have shown that, compared with standard laparoscopic or open TME, TaTME is a reasonable choice for treating of lower rectal cancer owing to its low conversion rate, similar postoperative complications, and good specimen quality (6). Laparoscopic-assisted TaTME (Lap-taTME) integrates the respective advantages of both peritoneal and transanal approaches, offering a balance that reduces surgical complexity. This approach shortens the learning curve of TaTME and has gradually become a mainstream alternative to TaTME. Surgeons have increasingly recognized the advantages of TaTME/Lap-taTME. However, it is not widely performed at present because of its different surgical concepts, operating paths, anatomical levels, high requirements for technology and equipment, high surgical difficulty, and long learning curve. Notably, Norway implemented a nationwide moratorium on TaTME due to challenges in rolling out the technology and the significant trend of local tumor recurrence observed to date (7).

In this study we asked if there is an operation capable of enhancing the exposure of the surgical field, thus mitigating the challenges of radical surgery for ultralow rectal cancer, while minimizing reliance on surgical equipment for broader applicability. We proposed a treatment combining transanal ISR with laparoscopic TME under direct view in the jackknife position for ultralow rectal cancer. The patient first assumed the jackknife position, and intersphincter resection was completed under a direct anal approach. A purse-string suture was then applied at the reserved end of the anal canal. Subsequently, the modified lithotomy position was used to complete the laparoscopic TME, and finally, the transanal anastomosis was completed in this position.

We believe that this procedure has the following advantages. First, using the horizontal line of the levator anal hiatus as a baseline, this approach optimally leverages the advantages of both transabdominal and transanal approaches, seamlessly combining the two by changing the patients’ position. High-quality resection of the terminal rectum was completed by close direct vision transanal ISR in the jackknife position, which reduced the difficulty of distal rectal resection by transabdominal ISR and the learning curve of TaTmeso/Lap-taTME by shortening the distance of transanal upward ionization. Second, transanal resection of the intersphincter under direct vision in the jackknife position reduces the need for laparoscopic instruments, constant pressure, and high-flow pneumoperitoneum. This approach also avoids complications such as carbon dioxide embolism and subcutaneous emphysema that may be caused by transanal endoscopy itself, promoting its development and widespread adoption. Third, with the advantage of the jackknife position, the operation is more convenient, the surgical field of view is clearer, and the surgical area is more fully exposed, which is conducive to high-quality completion of the operation. Finally, the tumor and distal rectum naturally sag under gravity in the jackknife position, so the positioning of the distal resection margin of rectal cancer is more accurate, avoiding unnecessary sacrifice of the distal rectal tube. To date, this procedure has not been previously reported in the literature. We believe that this operation is suitable for ultralow rectal cancer with a tumor distance of less than 3 cm from the anal margin.

TME is the gold standard for surgical treatment of low and middle rectal cancer. DRM and CRM are crucial to the quality of surgical resection and are closely related to patient prognosis (8). Denost (9) suggested that the upward movement of the tumor from the sphincter space through the anus could increase the distance of the tumor from the surgical surface, thereby reducing the local recurrence rate and increasing the negative rate of the circumferential incisal margin. Transanal ISR combined with laparoscopic TME directly entered the peripheral space of the mesocolium at the direct view of the jackknife position, and the distal incisal margin of the tumor was severed under direct view, so the safety of the subincisal margin and circumferential incisal margin of the tumor could be ensured. The mesocolium was completely resected in the 10 patients reported above, and the CRM and DRM were negative. The common complications after radical resection of ultra-low rectal cancer include anastomotic complications (anastomotic fistula, anastomotic bleeding, anastomotic stenosis), intestinal obstruction, infection, urinary retention, and deep vein thrombosis. One patient developed grade A anastomotic fistula 30 days after surgery, which healed after conservative treatment, 2 patients developed membranous anastomotic stenosis and were cured by anal enlargement. No significant postoperative complications were observed in the remaining patients. Low anterior resection syndrome is an inevitable complication after ISR surgery. Ito's studies have shown that the anal function of patients after ISR surgery will gradually improve and become stable within 6–12 months (10). Wexner and LARS scores showed that the patient did not have severe postoperative defecation incontinence, which may be related to the absence of neoadjuvant radiotherapy before surgery, clear anatomical structure during surgery, and appropriate extension of postoperative fistula restoration time.

Transanal Transection and Single-Stapled Anastomosis (TTSS) strategy is feasible, safe and leads to very low anastomotic leak rates after TME for rectal cancer (11, 12). This is a TTSS modified technique in which the key procedural steps remain similar, but the surgical sequence and positions are different. The sample size included in this report is relatively small, which may lead to large research bias and lack of evidence-based medicine. The operation was performed in a jackknife position followed by a modified lithotomy position, switching positions during surgrey will delay the operation. The operation followed a transanal and then transabdominal sequence, and abdominal exploration could not be performed first. Therefore, accurate staging must be performed with complete imaging examination before surgery. The postoperative follow-up time of patients is short, and the recurrence rate, long-term survival and quality of life of patients are still unclear. Further results will require multicenter randomized controlled trials with a larger number of cases and longer follow-up.

## Conclusions

We believe that our novel surgical approach of combining transanal ISR with laparoscopic TME is technically feasible and oncologically safe for treating patients with ultralow rectal cancer, and that postoperative bowel function following this procedure is acceptable. At the same time, we encourage more clinical centers to engage and participate actively in the study.

## Data Availability

The original contributions presented in the study are included in the article/Supplementary Material, further inquiries can be directed to the corresponding author.
